# Accuracy of ChatGPT in Neurolocalization

**DOI:** 10.7759/cureus.59143

**Published:** 2024-04-27

**Authors:** Waleed F Dabbas, Yousef M Odeibat, Mohammad Alhazaimeh, Mohammad Y Hiasat, Amer A Alomari, Ala Marji, Qais A Samara, Bilal Ibrahim, Rashed M Al Arabiyat, Ghena Momani

**Affiliations:** 1 Division of Neurosurgery, Department of Special Surgery, Faculty of Medicine, Al-Balqa Applied University, Al-Salt, JOR; 2 Department of Neurosurgery, Neuron Clinics, Amman, JOR; 3 Division of Neurosurgery, Department of Clinical Sciences, Faculty of Medicine, Yarmouk University, Irbid, JOR; 4 Department of Neurological Surgery, Neuron Clinics, Amman, JOR; 5 Department of Neurosurgery, San Filippo Neri Hospital/Azienda Sanitaria Locale (ASL) Roma 1, Rome, ITA; 6 Division of Neurosurgery, Department of Special Surgery, Faculty of Medicine, Mutah University, Al-Karak, JOR; 7 Department of Neurosurgery, King Hussein Cancer Center, Amman, JOR; 8 Department of General Practice, Al-Hussein Salt New Hospital, Ministry of Health, Al-Salt, JOR; 9 Faculty of Medicine, The Hashemite University, Zarqa, JOR

**Keywords:** anatomical localization, generative pre-trained transformers, anatomy, diagnosis, brain anatomy, neurolocalization, chatgpt, neurosurgery, neuroanatomy, artificial intelligence

## Abstract

Introduction

ChatGPT (OpenAI Incorporated, Mission District, San Francisco, United States) is an artificial intelligence (AI) chatbot with advanced communication skills and a massive knowledge database. However, its application in medicine, specifically in neurolocalization, necessitates clinical reasoning in addition to deep neuroanatomical knowledge. This article examines ChatGPT's capabilities in neurolocalization.

Methods

Forty-six text-based neurolocalization case scenarios were presented to ChatGPT-3.5 from November 6th, 2023, to November 16th, 2023. Seven neurosurgeons evaluated ChatGPT's responses to these cases, utilizing a 5-point scoring system recommended by ChatGPT, to score the accuracy of these responses.

Results

ChatGPT-3.5 achieved an accuracy score of 84.8% in generating “completely correct” and “mostly correct” responses. ANOVA analysis suggested a consistent scoring approach between different evaluators. The mean length of the case text was 69.8 tokens (SD 20.8).

Conclusion

While this accuracy score is promising, it is not yet reliable for routine patient care. We recommend keeping interactions with ChatGPT concise, precise, and simple to improve response accuracy. As AI continues to evolve, it will hold significant and innovative breakthroughs in medicine.

## Introduction

Just as HAL 9000 was entrusted with the critical functions of the spacecraft in 2001: A Space Odyssey movie, real-life artificial intelligence (AI) systems are being explored for their potential in the high-stakes arena of medicine. In the GPT-4 report by OpenAI, ChatGPT-3.5 achieved a 53% score, while ChatGPT-4 scored 75% in answering the Medical Knowledge Self-Assessment Program Exam [1]. In answering the questions of the Self-Assessment Neurosurgery (SANS) Exam, ChatGPT-4 scored 83.4%, surpassing the average user score of 72.8% [2]. Also, ChatGPT-4 scored 64% on the Specialty Certificate Examination (SCE) Neurology Web Question bank, higher than the 60.2% average score of all candidates [3].

However, unlike many other disciplines, medicine cannot rely on a tool that occasionally provides incorrect answers, even if such instances are rare [4]. Yet, the field of AI is an emerging and rapidly evolving field, and the future of AI is full of potential for unprecedented advancements and possibilities in medicine.

ChatGPT is an AI Chatbot. It is pre-trained on a massive database and possesses advanced human-like communication abilities. However, in the context of medical applications, ChatGPT requires the ability to do clinical reasoning. Neurolocalization demands thorough neuroanatomical knowledge paired with the art of clinical reasoning. In this article, we explore ChatGPT’s capabilities in neurolocalization.

## Materials and methods

We designed 46 neurolocalization case scenarios to be brief, focused, and direct. Given ChatGPT’s text-only input capabilities, the cases were structured to be text-based without the necessity for radiological image input. Each case is structured to have one possible definitive answer. Six cases necessitated a subtle hint of radiological finding to narrow the differential to a singular definitive answer (e.g., ‘unremarkable lumbar MRI', 'left skull base mass on brain MRI', 'cortical lesion on brain MRI', 'presentation with stroke'). Each question concluded with the statement: “What is the most likely neurolocalization in this patient's condition?”.

The cases were submitted to ChatGPT-3.5 from November 6th, 2023, to November 16th, 2023. Each case was communicated to ChatGPT in a distinct chat session. The first response provided was utilized for examination.

For each case, a structured file has been created, which includes the case scenario, ChatGPT’s generated response, and a suggested standardized answer for comparison and evaluator guidance. Evaluators were given the flexibility to deviate from the suggested answer if they thought another answer was more appropriate for the case. Subsequently, these files were emailed to seven neurosurgery specialists for evaluation.

We developed the scoring system based on a recommendation from ChatGPT (Table 1). We asked ChatGPT to suggest a 5-point scale to assess its accuracy.

**Table 1:** 5-point accuracy scoring system derived from ChatGPT's recommendation.

Score	Category	Description
5	Completely Correct	The response is factually accurate, relevant, and comprehensive, fully addressing the question or request.
4	Mostly Correct	The response is generally accurate and relevant to the question or request, but it may contain minor errors or omissions.
3	Partially Correct	The response provides some accurate information but also includes inaccuracies or misses important context.
2	Mostly Incorrect	The response contains inaccuracies or misunderstands the context, but it still has some elements of correctness.
1	Completely Incorrect	The response is factually inaccurate or nonsensical. It does not address the question or request at all.

## Results

ChatGPT-3.5 achieved an accuracy score of 84.8% in generating “completely correct” and “mostly correct” responses for 46 neurolocalization cases (Table 2). The mean score was 4.5 (SD 0.97), falling between “mostly correct” and “partially correct”. The median score was 5 (IQR: [4-5]), indicating limited variability in responses between “partially correct” and “mostly correct” (Table 3).

**Table 2:** Accuracy scores for ChatGPT's responses. Categorization of ChatGPT-3.5’s response accuracy into five distinct scores, ranging from "Completely Correct" to "Completely Incorrect". The assessment is based on 46 neurolocalization case responses (N=46), with the number of responses and respective percentages listed for each accuracy score.

Score Category	Number of ChatGPT’s Responses (N=46)
Completely Correct	32 (69.6%)
Mostly Correct	7 (15.2%)
Partially Correct	4 (8.7%)
Mostly Incorrect	2 (4.3%)
Completely Incorrect	1 (2.2%)

**Table 3:** Neurolocalization case topics and the scores of ChatGPT’s responses. The accuracy scores of 46 neurolocalization cases presented to ChatGPT-3.5, covering different anatomical structures, are scored on a five-point scale: 5: Completely Correct; 4: Mostly Correct; 3: Partially Correct; 2: Mostly Incorrect; 1: Completely Incorrect.

Case Number	Topic	Score	Case Number	Topic	Score
1	Optic Chiasm and Pituitary	5	24	Hypothalamus	4
2	Jugular Foramen	5	25	Cauda Equina	5
3	T4 Spinal Cord Segment	4	26	Ulnar Nerve at Guyon Canal	5
4	Abducent Nerve	5	27	Rostral Dorsal Midbrain	5
5	Hypothalamus	2	28	Fornix	5
6	Bilateral Occipital Lobes	5	29	Pones	3
7	Frontal Lobe	4	30	Lateral Medulla	5
8	Left Parietal Lobe	5	31	Arcuate Fasciculus	5
9	Hand Area of Precentral Gyrus	5	32	C5 Nerve Root	5
10	Thalamus	4	33	Peroneal Nerve	5
11	Right Parietal Lobe	5	34	Cervical Spinal Cord	5
12	Medial Precentral Gyrus	2	35	Midbrain	4
13	Temporal Lobe	5	36	Basal Ganglia	5
14	Ilioinguinal, Iliohypogastric, and Genitofemoral	4	37	Broca’s area	5
15	Cervical Spinal Cord	5	38	Pones	5
16	Hippocampus	5	39	Medulla	3
17	Pones	5	40	Lateral Femoral Cutaneous Nerve	4
18	Cerebellum	5	41	Axillary Nerve	5
19	Supplementary Motor Area	1	42	Facial Nerve	5
20	Neuromuscular Junction	5	43	Occipital Nerve	5
21	Sensory Cortex	5	44	Vestibulocochlear Nerve	5
22	Fusiform Gyrus	5	45	Abducent Nerve and Sympathetic Fibers in the Cavernous Sinus	3
23	Right Medical Longitudinal Fasciculus	3	46	Mandibular Division of Trigeminal Nerve	5

Seven neurosurgeons assessed the accuracy of ChatGPT’s responses, each evaluating 6 to 7 responses. The means of scores assigned by evaluators ranged from 4 to 5 (Table 4). An ANOVA analysis was conducted to determine if there were any significant differences in the scoring patterns among the evaluators. The analysis revealed an F-value of 0.75 (p-value 0.61), indicating no statistically significant difference in the means of scores assigned by evaluators, suggesting a consistent scoring approach.

**Table 4:** Each evaluator's total number of assessed responses and their respective mean of assigned scores. Each evaluator reviewed 6-7 ChatGPT responses. The combined mean score for all 46 responses was 4.5, with a standard deviation of 0.97.

Evaluator	Number of Evaluated Responses	Mean of Assigned Scores
1	7	4
2	7	4.3
3	7	4.7
4	7	4.6
5	6	4.5
6	6	4.2
7	6	5

ChatGPT processes text length in tokens. The longest case scenario had 102 words (126 tokens) and the shortest 28 words (34 tokens). The mean token count was 69.8 (SD 20.8). The Pearson correlation coefficient between cases’ token count and the assigned scores was -0.02 (p-value 0.89), indicating no meaningful statistical correlation between the token count and the assigned score within the observed range of cases’ token count. Appendix Figures 1, 2 show examples of the study cases asked to ChatGPT-3.5 and its responses.

## Discussion

Natural language processing (NLP)

The field of NLP is a domain of computational linguistics that focuses on equipping computers with the capacity to understand and manipulate human language [5]. Generative pre-trained transformers (GPTs) are advanced NPL models that utilize transformer technology, a form of a neural network model engineered to comprehend word meanings by establishing semantic relationships between words [6-7]. After pre-training the model with a massive amount of data, it acquires the ability to generate human-like responses [8] by predicting the next word in a text sequence, using the preceding words as input for that prediction [9].

GPT model evolution

The first GPT model, GPT-1, was introduced by OpenAI in June 2018. This model was pre-trained using the BooksCorpus dataset, which contains over 7,000 unique unpublished books [10] comprising approximately 5 gigabytes (GB) of data and equipped with 117 million parameters [11]. Parameters are internal settings or rules the model learns from data; they determine how the model processes and generates text, thereby fine-tuning the model’s performance.

In 2019, GPT-2 was introduced, incorporating extensive 1.5 billion parameters. It underwent pre-training using the WebText dataset, which includes more than 8 million documents, totaling 40 GB of text [12].

GPT-3 was released in 2020, featuring a staggering 175 billion parameters. It harnessed a substantial pre-training dataset of 45 terabytes of compressed plaintext before ﬁltering, and 570 GB after ﬁltering. The datasets used for pre-training included Common Crawl dataset, an expanded version of the WebText dataset, two internet-based books corpora (Books1 and Books2), and English-language Wikipedia [13].

In 2022, the first model of ChatGPT, ChatGPT-3.5, was released. It is designed and trained specifically to engage in conversational interactions based on a fine-tuned GPT-3.5 [14]. The most recent update to their knowledge base was in January 2022. In March 2023, the most advanced models, GPT-4 and ChatGPT-4, were released [1]. Unofficial estimations indicate that these models are empowered with a massive 1.7 trillion parameters [15]. The latest update to their knowledge was in December 2023

Neurolocalization

Neurolocalization necessitates a comprehensive grasp of neuroanatomy, functional neurological structures, and their interrelationships. ChatGPT-3.5 defined neurolocalization as “the process of identifying the specific anatomical location within the nervous system that is associated with particular neurological symptoms or signs. It involves pinpointing the area of the brain, spinal cord, or peripheral nerves responsible for a patient's neurological dysfunction. This process is crucial in diagnosing and treating neurological conditions accurately." Neurosurgery [16] and neurology [17] are perceived to be challenging medical disciplines, a view primarily attributed to the complexities involved in neuroanatomy and neurological localization. This raises an intriguing question regarding the performance of AI in these subjects.

Galetta et al. investigated the neurological localization and diagnostic capabilities of ChatGPT-4 [18]. They presented clinical cases to ChatGPT, engaging in a multi-step process where they initially posed cases and then refined their queries. Localization based on clinical history and examination exhibited an accuracy of 59% with easier cases compared to 33% with harder cases. A stepwise introduction of ancillary clinical data improved the localization accuracy for easier cases to 67% but paradoxically worsened the answers for harder cases to localize 0% accurately. However, when ChatGPT was provided with history, exam, and ancillary data in a single step, its performance substantially improved, accurately localizing 93% of easier cases and 78% of harder cases.

Our investigation

Our study examined ChatGPT-3.5, considering its potential to attract a broader audience compared to ChatGPT-4, as it offers free access. We designed our cases to be brief and focused, offering one potential answer to simplify the assessment process. Our cases were text-based, given ChatGPT’s limitation in processing images. Nevertheless, in six cases, we had to offer ChatGPT with hints related to radiological findings to narrow the scope of neurolocalization to a single, definitive response. To assess response accuracy, we adopted a scale proposed by ChatGPT itself after requesting it to suggest a 5-point scale to score the accuracy of ChatGPT responses.

We examined 46 neurolocalization cases asked to ChatGPT-3.5. The accuracy rate was 84.8% for “mostly accurate” or “completely accurate” responses. While this accuracy rate is promising for the future of AI in the medical field, it is still not sufficiently reliable for patient care. Thus, until the present time, the use of ChatGPT in medicine is still limited to a potential supplementary tool in decision-making processes [19] under the careful supervision of experienced medical professionals, much like other tools that physicians use to aid in patient care.

ChatGPT consistently performs analyses of given case scenarios. Although it may not consistently achieve a perfect score of 5 (indicating a “completely correct” response), it demonstrates logical reasoning in its responses. ChatGPT also understands basic neurological scoring systems, such as The Glasgow Coma Scale (GCS) and The Medical Research Council (MRC) Scale. Moreover, it consistently recommends further investigations and advises seeking proper medical consultation from a real doctor.

In one response, the evaluator found ChatGPT's response more accurate than the suggested answer. Reasons for assigning “mostly correct” and “partially correct” scores included: not specifying or incorrectly specifying the side of the lesion, prioritization of non-neurological differentials over neurological localization, and providing conclusions that were incomplete or inaccurate but based on logical reasoning. One response scored “completely incorrect”, a case of postoperative supplementary motor area syndrome. Additionally, two responses were “mostly incorrect”, addressing the neurolocalization of the hypothalamus for gelastic seizure and the medial precentral gyrus for lower limb monoplegia. 

ChatGPT and neurosurgery

It is evident from the GPT-4 technical report that OpenAI has a focus on medical applications; it reported ChatGPT-4 scoring 75% on the Medical Knowledge Self-Assessment Program exam, a notable improvement from ChatGPT-3.5's score of 53% [1]. Furthermore, multiple studies have investigated the accuracy of ChatGPT in answering questions related to neurology and neurosurgery.

Chen et al. reported that ChatGPT correctly answered 65.8% of 509 Neurology board-style examination questions on the first attempt and 75.3% over three attempts, comparable to the 26th and 50th percentiles of human test-takers, respectively [20]. In the study by Giannos [3], ChatGPT 3.5 Legacy scored 42%, ChatGPT 3.5 scored 57%, and GPT-4 scored 64% on the Neurology SCE Question Bank, while the 2022 pass rate for UK trainees was 79.6%, and for all candidates was 60.2%.

Guerra et al. examined ChatGPT-4, correctly answering 76.6% (453/591 questions) of the SANS Exam [21]. When limited to text-based questions, its accuracy improved to 79% (377/477 questions), outperforming the mean of test-takers (69.3%), neurosurgery residents (61.5%), and medical students (26%). Also, in a study by Ali et al., ChatGPT-3.5 correctly answered 73.4% (95% CI: 69.3%-77.2%) (367/500 questions) of SANS Exam questions, while ChatGPT-4 correctly answered 83.4% (95% CI: 79.8%-86.5%) (417/500 questions) [2]. In comparison, users' mean score was 72.8% (95% CI: 68.6%-76.6%). Liu et al. found that ChatGPT-3.5 aligned with neurosurgical guidelines in 42% (21/50 questions) of responses, while ChatGPT-4 achieved 72% (36/50 questions) accurate responses [22]. 

How to use ChatGPT

AI is not meant to replace medical professionals, but in the near future, physicians who know how to use AI will probably replace those who do not [23]. Consequently, it is essential to consider several key points while engaging with ChatGPT:

1) Keep it concise: always consider ChatGPT’s limited context window. If this limit is reached, earlier parts of the conversation are "forgotten" to make room for new input. ChatGPT-3.5 can handle 4,096 tokens (approximately 3,000 words), and ChatGPT-4 up to 8,192 tokens (approximately 6,000 words). This token count includes the user’s input and the generated responses [24].

2) Keep it clear and precise: users can guide ChatGPT to generate more accurate responses by formulating clear and direct inputs [25]. Conversely, ambiguous or incomplete inputs may lead to less accurate or relevant responses [26].

3) Keep it simple: balancing input complexity prevents ChatGPT from being confused and overloaded with information and increases the likelihood of generating a more accurate response [18].

ChatGPT’s limitations

AI has a promising future in medical applications, and ChatGPT is the first step in this long journey. While ChatGPT can assist in decision-making and reduce the workload for neurosurgeons [27], it is not yet equipped to handle real-life cases with complex, intertwining variables where the superior judgment of the human brain is evident. Additionally, an inherent limitation of ChatGPT is its inability to perform physical examinations or process radiological images [28]. Furthermore, an intriguing question arises: how will we, as beings driven by social and emotional needs, form connections and interact with these machines operating on ones and zeros? [29].

Limitations

Firstly, while our use of concise case scenarios facilitated the assessment of ChatGPT’s response, it is important to acknowledge that these simplified scenarios may not fully capture the complexity of real-world neurological cases. This discrepancy could affect the generalizability of our findings to more real-life situations. Secondly, our study employed ChatGPT-3.5 instead of the more advanced ChatGPT-4. This decision was based on the free access provided by ChatGPT-3.5, which attracts a broader user base. However, this choice may have implications for the study's applicability, as ChatGPT-4 potentially offers enhanced capabilities and improved accuracy.

## Conclusions

ChatGPT-3.5 achieved an accuracy score of 84.8% in generating “completely correct” and “mostly correct” responses for 46 neurolocalization cases. While this accuracy score is promising, it is not yet reliable for routine patient care. We advise keeping interactions with ChatGPT concise, precise, and simple to improve response accuracy. As AI continues to evolve, it will hold significant and innovative breakthroughs in medicine.

## Figures and Tables

**Figure 1:**
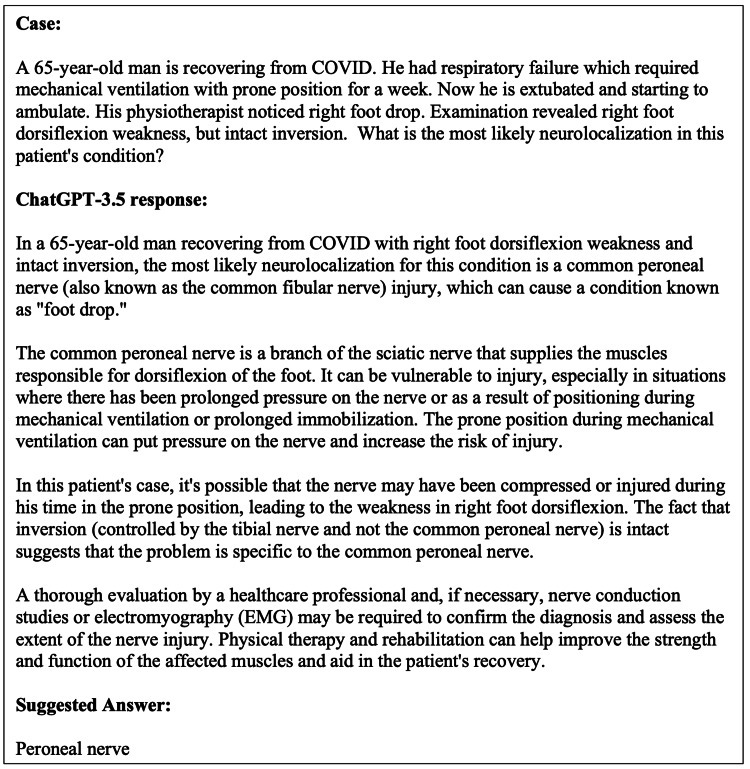
Neurolocalization case study of peroneal nerve injury presented to ChatGPT-3.5, featuring the examined prompt, ChatGPT's response, and the suggested answer. ChatGPT's response starts with a summary of the case and provides an answer that was scored as "completely correct" by the evaluator. It subsequently covers the relevant anatomy and pathophysiology, explains how other differential diagnoses were excluded, and concludes by advising further evaluation by a healthcare professional, recommending additional diagnostic tests, and discussing potential treatment options.

**Figure 2:**
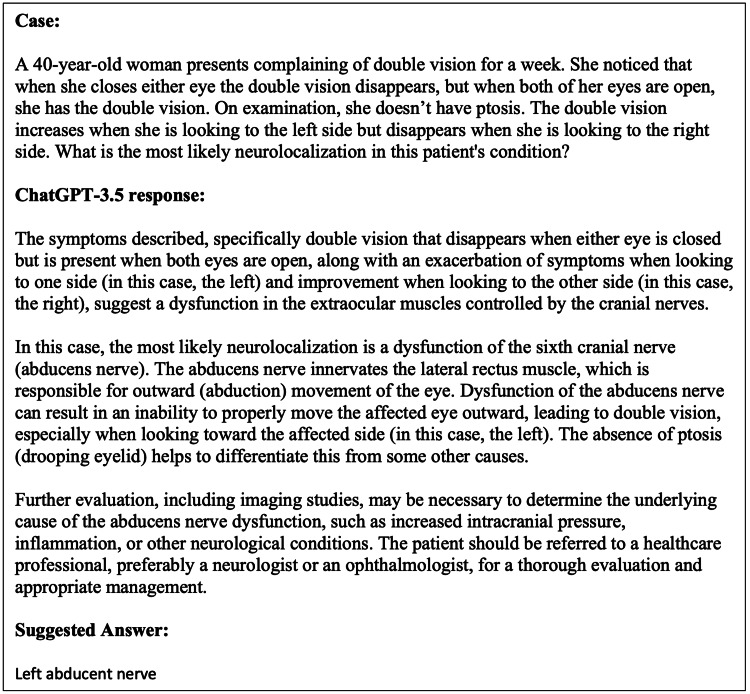
Neurolocalization case study of abducent nerve palsy presented to ChatGPT-3.5, featuring the examined prompt, ChatGPT's response, and the suggested answer. ChatGPT identifies and prioritizes critical information from the neurolocalization case study prompt to construct its response.
